# Cecal volvulus in malrotation: a rare postpartum complication—a case report

**DOI:** 10.1093/jscr/rjaf225

**Published:** 2025-04-25

**Authors:** Asratu Getnet Amare, Cheru Lilay Gebrehiwet, Leaynadis Kassa Lake, Muluken Assefa Zemariam, Michael A Negussie, Melkamu Temesgen Moges

**Affiliations:** Department of Surgery, School of Medicine, College of Medicine and Health Sciences, University of Gondar, Maraki Street, Gondar City, Central Gondar Zone, PO Box 196, Gondar, Ethiopia; Department of Surgery, School of Medicine, College of Medicine and Health Sciences, University of Gondar, Maraki Street, Gondar City, Central Gondar Zone, PO Box 196, Gondar, Ethiopia; Department of Surgery, School of Medicine, College of Medicine and Health Sciences, University of Gondar, Maraki Street, Gondar City, Central Gondar Zone, PO Box 196, Gondar, Ethiopia; Department of Surgery, School of Medicine, College of Medicine and Health Sciences, University of Gondar, Maraki Street, Gondar City, Central Gondar Zone, PO Box 196, Gondar, Ethiopia; School of Medicine, College of Health Sciences, Addis Ababa University, Tikur Anbessa Specialized Hospital, Churchill Avenue, Lideta Sub-City, PO Box 5657, Addis Ababa, Ethiopia; Department of Surgery, School of Medicine, College of Medicine and Health Sciences, University of Gondar, Maraki Street, Gondar City, Central Gondar Zone, PO Box 196, Gondar, Ethiopia

**Keywords:** cecal volvulus, malrotation, postpartum, hemicolectomy, case report

## Abstract

A cecal volvulus in malrotation in a postpartum patient is an infrequent clinical entity. We present a case of a 24-year-old male patient with cecal volvulus in malrotation. The patient underwent counterclockwise detorsion, division of Ladd’s bands, right hemicolectomy with end ileostomy, transverse colopexy, and early ileostomy reversal. This case highlights the challenges in diagnosing and managing these rare conditions.

## Introduction

Cecal volvulus is relatively uncommon and involves the rotation of the cecum, terminal ileum, or ascending colon [1]. It accounts for less than 2% of all intestinal obstructions [2] and approximately 10%–60% of all colonic volvulus cases [3]. Cecal volvulus occurs when a hypermobile cecum twists around its mesenteric pedicle [4]. Congenital intestinal malrotation is a complex disorder caused by faulty rotation and fixation of the gut during the 5th to 11th weeks of fetal life [2, 3]. This case report describes a rare presentation of cecal volvulus associated with intestinal malrotation in an adult postpartum patient.

## Case presentation

A 24-year-old para-1 mother was referred to our hospital 1 day after a vaginal delivery for further evaluation and management. Two days before delivery, she developed peri-umbilical crampy abdominal pain, which was later accompanied by bilious vomiting and abdominal distension. She had not passed flatus or had a bowel movement for the past day. She denied any fever, urinary symptoms, or history of prior abdominal surgery.

**Figure 1:**
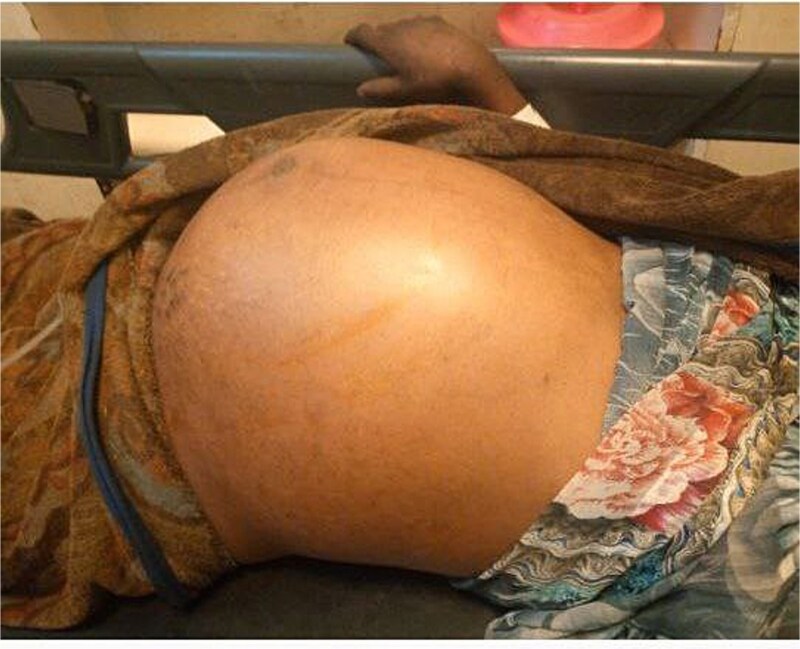
Photograph showing a grossly distended abdomen postpartum in the patient.

On examination, she appeared acutely ill. Her vital signs showed a blood pressure of 90/60 mmHg, a pulse rate of 120 beats per minute (tachycardia), and a temperature of 36.6°C. Her oxygen saturation was 96% on room air. Abdominal examination revealed a grossly distended abdomen (Fig. 1) that was hyper-tympanic to percussion with diffuse tenderness. The uterus was consistent with a 12-week gravid size. A digital rectal examination revealed stool in the rectum.

**Figure 2:**
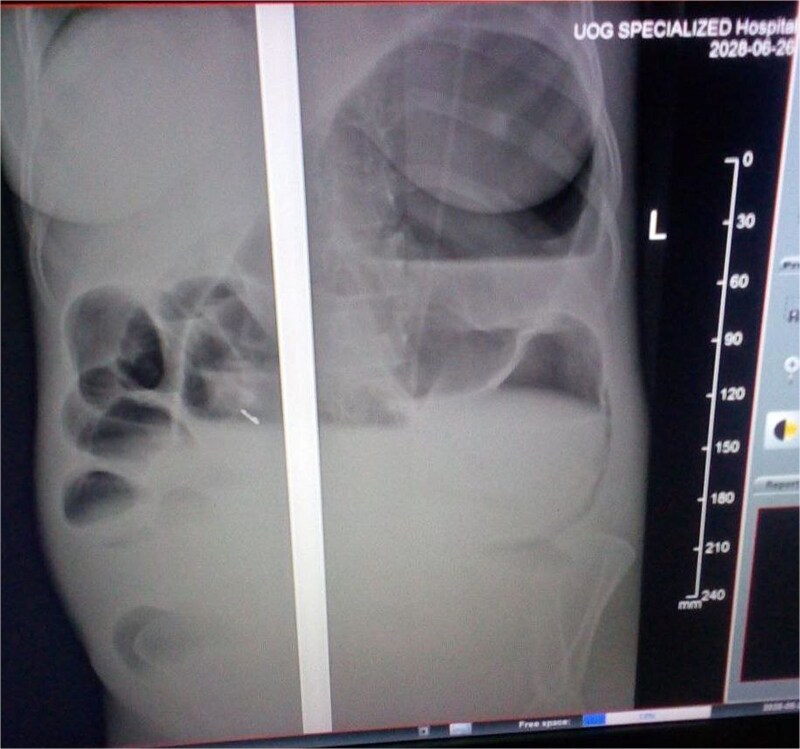
Abdominal X-ray showing the dilated colon and multiple air-fluid levels.

Laboratory investigations, including complete blood count (CBC), electrolytes, and organ function tests, were within normal ranges. A plain abdominal X-ray demonstrated bowel loops extending from the right lower quadrant to the left upper quadrant, with a paucity of air in the distal segment and peripheral air-fluid levels (Fig. 2). A differential diagnosis of large bowel obstruction, possibly secondary to gangrenous cecal volvulus, was considered.

Her urinary bladder was catheterized, and a nasogastric tube was inserted. She was resuscitated with intravenous fluids and administered intravenous antibiotics.

**Figure 3:**
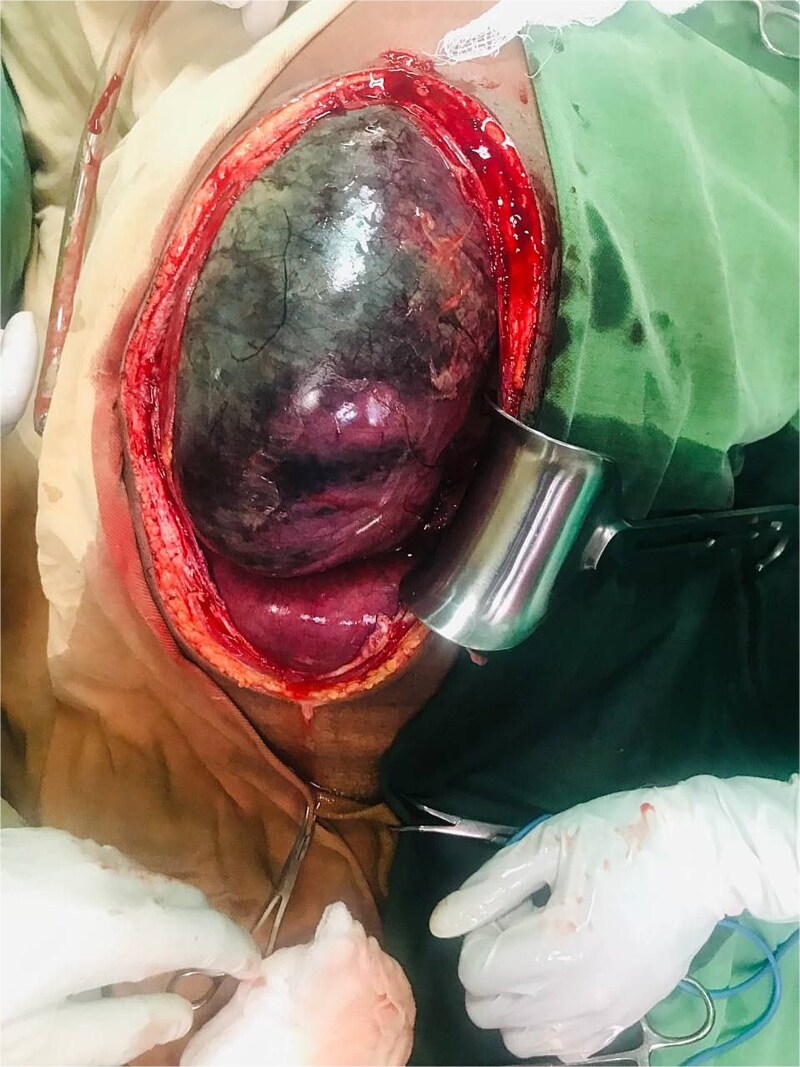
Dilated, gangrenous cecum twisted with descending colon and distal ileum.

An emergency exploratory laparotomy was performed, revealing a distended small bowel on the right side and a distended large bowel on the left side of the abdomen. The cecum was dilated, gangrenous, and twisted in a counterclockwise direction, involving the descending colon and distal ileum (Fig. 3).

**Figure 4:**
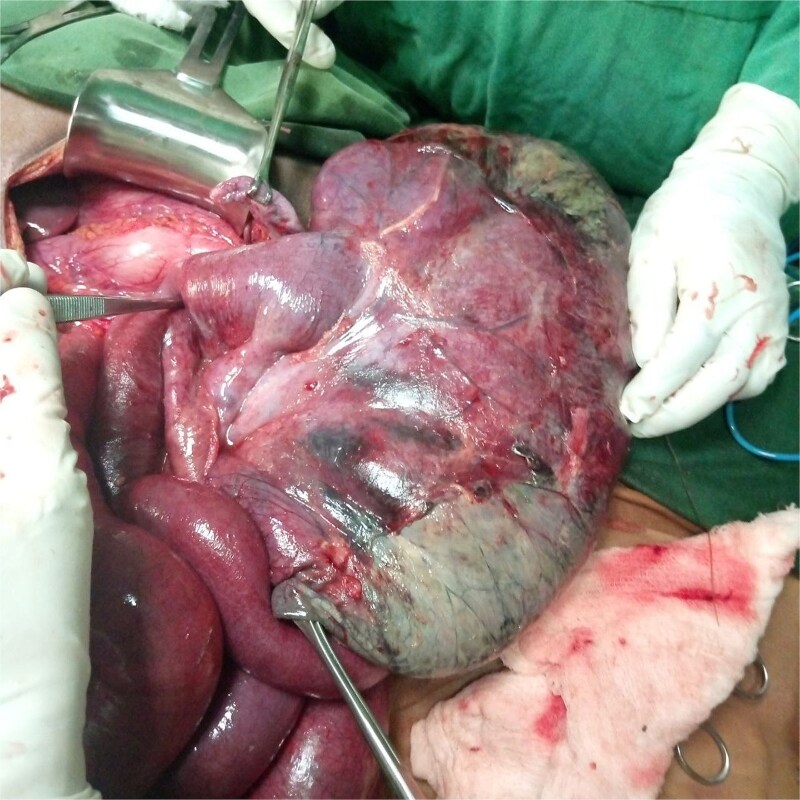
Picture showing an elongated appearance of the stomach.

The cecum and ascending colon were not located in the right gutter and were freely mobile, similar to the transverse colon. The duodenum did not cross the midline, and bands were observed over the duodenum and between loops of the large bowel. The stomach appeared elongated (Fig. 4).

**Figure 5:**
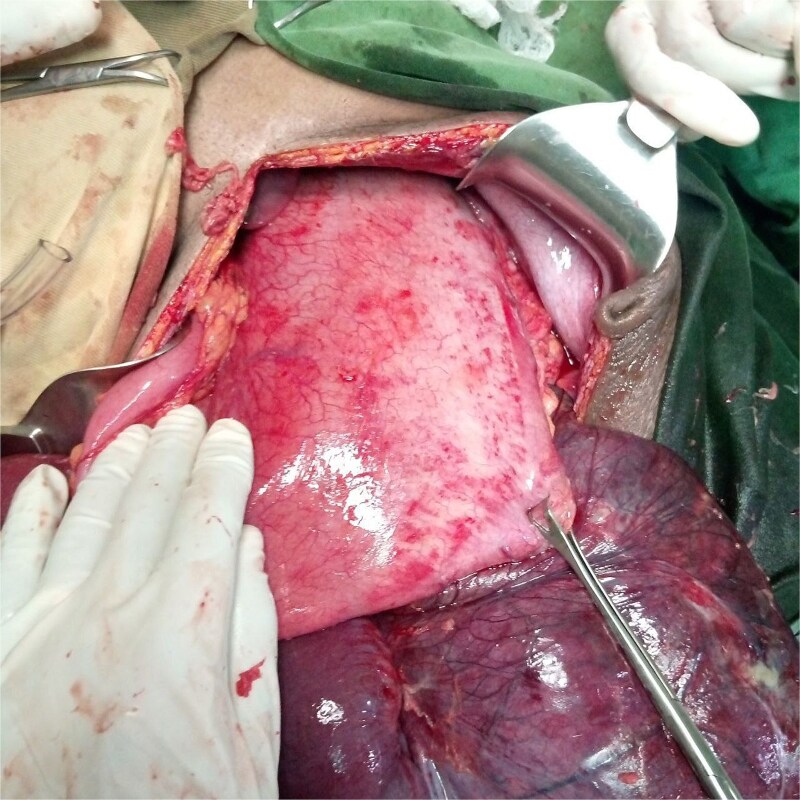
Picture of cecum following detorsion.

During the procedure, the cecum was decompressed of intestinal air using a simple cannula inserted through the tenia, followed by detorsion (Fig. 5). Ladd’s bands were divided, and a right hemicolectomy, including appendectomy, was performed along with the creation of an end ileostomy.

Intraoperatively, the patient developed shock, necessitating the initiation and escalation of a low dose of noradrenaline. She was transferred to the surgical intensive care unit for further management. Within 24 h postoperatively, her shock resolved, noradrenaline was tapered and discontinued, and she was extubated. By the second postoperative day, she was moved to the recovery unit.

On the sixth postoperative day, she developed a high-output stoma, necessitating an ileostomy reversal. She recovered well and was discharged on the tenth postoperative day. At her 1-month follow-up, she tolerated oral feeds well without abdominal pain or distension. By her second postoperative month, she remained stable, with no reported complications.

## Discussion

Cecal volvulus in the setting of malrotation in a postpartum patient is a rare presentation. First described by Rokitansky in 1837 as a form of intestinal strangulation, cecal volvulus typically arises from cecal hypermobility combined with precipitating factors such as a colonic tumor, abdominal mass, pregnancy, or congenital conditions (e.g. Ehlers-Danlos syndrome). Cecal volvulus during pregnancy has been reported in several publications, with an incidence ranging from 1 in 2500 to 1 in 3500 [5].

Patients suspected of having a cecal volvulus should undergo appropriate investigations, including colonoscopy, barium enema, abdominal computed tomography scan, or surgery [6].

Gut malrotation is a rare condition that arises during fetal development due to incomplete intestinal rotation around the superior mesenteric pedicle. The majority of intestinal malrotation cases present within the first 2 weeks of life [7]. It is uncommon for malrotation to manifest in adulthood [8]. In most reported cases, an accurate diagnosis is made during surgery [9], as in our report.

Surgery is the primary management strategy for cecal volvulus and can be performed electively or in emergencies [10, 11].

Surgical options include open or laparoscopic approaches. Nonoperative interventions, such as enema or colonoscopic reduction, have been attempted but are associated with high rates of recurrence and failure [12].

In cases of complicated cecal volvulus involving ischemia, gangrene, or perforation, endoscopic reduction is contraindicated, and surgery becomes mandatory. When bowel necrosis, gangrene, or perforation is present, surgical resection is performed [13].

The surgical options include bowel resection with primary anastomosis, cecopexy, or cecostomy, depending on intraoperative findings and the patient’s hemodynamic stability [14]. Cecopexy is recommended for cecal volvulus with viable bowel and is associated with a recurrence rate of 8.8% and a morbidity rate of 0%–8%. Detorsion and cecopexy can be performed laparoscopically or with open approaches [15].

The Ladd’s procedure is commonly used to treat midgut malrotation. It typically involves dividing the Ladd’s bands that overlay the duodenum, widening the narrowed root of the small bowel mesentery, dividing adhesions around the superior mesenteric artery, counterclockwise detorsion of the midgut, and performing an appendectomy [16]. Originally described for pediatric patients, not all components of the classic Ladd’s procedure may be applicable in adults.

In our patient with cecal volvulus, the Ladd’s bands were divided, and a right hemicolectomy (including appendectomy), ileal resection, and end ileostomy were performed, along with counterclockwise detorsion of the midgut. A cecopexy was not performed due to ischemia of the cecum and the associated risk of colonic perforation, which can lead to significant morbidity and mortality. Following bowel resection and primary anastomosis, a transverse colopexy was performed to reduce the risk of recurrent colonic volvulus. Transverse colopexy has been documented as a viable treatment option in acute transverse colon volvulus [6].

## Conclusion

Cecal volvulus requires a high index of suspicion in pregnant women with acute abdomen. Early diagnosis prevents serious complications.
